# Health Data Linkage for UK Public Interest Research: Key Obstacles and Solutions

**DOI:** 10.23889/ijpds.v4i1.1093

**Published:** 2019-04-02

**Authors:** Miranda Jane Mourby, James Doidge, Kerina H Jones, Stergios Aidinlis, Hannah Smith, Jessica Bell, Ruth Gilbert, Peter Dutey-Magni, Jane Kaye

**Affiliations:** 1 Centre for Health, Law and Emerging Technologies, University of Oxford, Ewert House, Oxford, OX2 7DD, UK; 2 Great Ormond Street Hospital Institute of Child Health and UCL Institute of Informatics, 222 Euston Road, London NW1 2DA, UK; 3 Intensive Care National Audit & Research Centre, 24 High Holborn, London WC1V, UK; 4 Data Science Building, Swansea University Medical School, Singleton Park, Swansea SA2 8PP, UK; 5 Centre for Health, Law and Emerging Technologies, Melbourne Law School, Level 9, 185 Pelham Street, University of Melbourne, Victoria 3010, Australia

## Abstract

**Introduction:**

Analysis of linked health data can generate important, even life-saving, insights into population health. Yet obstacles both legal and organisational in nature can impede this work.

**Approach:**

We focus on three UK infrastructures set up to link and share data for research: the Administrative Data Research Network, NHS Digital, and the Secure Anonymised Information Linkage Databank. Bringing an interdisciplinary perspective, we identify key issues underpinning their challenges and successes in linking health data for research.

**Results:**

We identify examples of uncertainty surrounding legal powers to share and link data, and around data protection obligations, as well as systemic delays and historic public backlash. These issues require updated official guidance on the relevant law, approaches to linkage which are planned for impact and ongoing utility, greater transparency between data providers and researchers, and engagement with the patient population which is both high-profile and carefully considered.

**Conclusions:**

Health data linkage for research presents varied challenges, to which there can be no single solution. Our recommendations would require action from a number of data providers and regulators to be meaningfully advanced. This illustrates the scale and complexity of the challenge of health data linkage, in the UK and beyond: a challenge which our case studies suggest no single organisation can combat alone. Planned programmes of linkage are critical because they allow time for organisations to address these challenges without adversely affecting the feasibility of individual research projects

## Highlights

- Linkage of health data and other forms of public sector data in the UK is an under-utilised resource for public interest research.
- We use three UK case studies to examine the obstacles which prevent this area of research from reaching its full potential.
- Recommendations are made in respect of each obstacle, with an emphasis on planned programmes of linkage.
- This should allow data providers time to address the various governance issues which will inevitably arise in health data linkage.

## Introduction

Analysis of linked health data across national or local populations has enormous public interest potential. With its capacity to reveal issues in patient pathways, or trends in mortality which could be addressed through state intervention, it is not an overstatement to say that data can save lives [1]. Yet even when the stakes are high, obstacles both legal and practical in nature can prevent potentially life-preserving work:

### Example 1 (Drawn from SAIL Case Study)

In 2010 the Chief Medical Officer for Wales requested research into the factors underlying the higher mortality rates recorded during winter. This research required access to anonymised individual- and household-level data from the Valuation Office Agency (an agency of HM Revenue and Customs), linked with health data from the Secure Anonymised Information Linkage Databank (‘SAIL’). In their Briefing on what was to become the Digital Economy Act 2017 or ‘DEA’ as we shall refer to it in this paper, the Royal Statistical Society noted as follows:

*‘The request was submitted in April 2010, and legal teams discussed appropriate ways of providing the data. In February 2012 HMRC confirmed that they believed they were unable to share the data due to statutory constraints on data sharing. This had a negative impact on the implementation of government policy that might have had an impact on mortality* [2].’

The above example was published in support of a single statutory power for public authorities to share data for research in the DEA. There was already evidence that the framework of laws allowing public authorities to share data for research was too complicated [3], and it was thought a single generic power to share data for research purposes would prevent this kind of impasse for research.

This power was duly introduced in the DEA, passed in 2017 and brought into force in May 2018 (with additional time required to set up a researcher accreditation system). The type of data in question—information collected by public authorities as part of their day-to-day functions—is often referred to in policy and research circles as ‘administrative data.’ The term is not used within any legislation, and there is no consistently used terminology for information about citizens collected in the course of delivering health and other public services. We use the term as the most useful way of referring to this broad, overarching category of state-assembled information.

Administrative data are typically used in research in an ‘anonymised’ form to provide information about populations, not individuals. We will explore the term ‘anonymisation’ in a subsequent section, but essentially ‘anonymised’ data consist of information which has been processed and protected in such a way that it no longer relates to an identifiable individual. This is the meaning of the term under UK and European data protection law. The Administrative Data Research Network (‘ADRN’) was set up with investment from the Economic and Social Research Council (‘ESRC’) in 2013 to facilitate researcher access to anonymised administrative data [4]. Senior members of the ADRN were involved in drafting the research provisions in the DEA [5]. Even before this new statutory power was brought into effect, the following second example illustrates how the ADRN helped to facilitate access to administrative data:

### Example 2 (Drawn from ADRN & SAIL Case Studies)

This project analysed the Welsh Warm Homes Nest Scheme, which offered eligible householders free home energy efficiency improvements such as new boilers, central heating or insulation. A key focus of the scheme was to promote the health of those who could not afford such measures, particularly during winter.

Health data from SAIL were linked with dwelling level data indicating recipients of the scheme, before being accessed in anonymised form by the researchers through the Administrative Data Research Centre in Wales (part of the ADRN). The researchers were then able to compare a control group who were eligible for the fuel efficiency measures but who had not yet received them, with a group of householders who had received the measures. A final linked dataset with the respiratory events of 16,353 recipients, as well as of 24,895 people in the control group, was analysed and compared with the respiratory events in the winter before.

The initial report concluded that further analysis was required to establish whether the differences in health events between the recipient and control groups observed at baseline could be attributed to successful targeting of the scheme [6]. However, the 16,353 fuel efficiency recipients experienced a 3.9% decrease in GP recorded respiratory events in the winter after the installation (and a 6.5% decrease in recorded asthma events). The control group, by contrast, saw an increase in GP recorded respiratory events in the winter after (a 9.8% rise for respiratory events, and 12.5% increase for asthma events).

This second example can be seen as a positive counterpart to the first, and perhaps even a sign of progress. Nevertheless, concerns remain as to the future of health data linkage and access for research. Data held by public authority health service providers (which includes National Health Service – or ‘NHS’– patient data) were excluded from the data sharing powers under s.64 DEA. It therefore remains unclear how such NHS data, which we refer to in this paper as ‘health data’, can lawfully be linked with other public sector information, even in cases of demonstrable public interest.

The concept of ‘public interest’ is inevitably broad, and potentially controversial. For the purposes of this paper, however, we use the term in the meaning bestowed to it for the purposes of the DEA, as this is the purpose for which health data could have been used had they been included within research data sharing powers in the new Act. By ‘public interest’, we therefore mean (inter alia) research whose primary purpose is to provide evidence for policy evaluation, guide critical decision making, significantly extend understanding of social trends or replicate, validate or challenge existing research (including official statistics), as these are all included in a non-exhaustive list of ‘public interest’ features of research for which data may be shared under the DEA [7].

While the Digital Economy Act framework has not provided a definitive articulation of ‘public interest’, it has carved out categories of research of sufficient public importance to justify the use of de-identified administrative data. Crucially, it also allows such data to be linked with other information prior to de-identification disclosure for research purposes, and thus provides a gateway for research using linked administrative data, on which we focus in this paper. While s.64 DEA does not explicitly sanction linkage, and merely refers to ‘processing’ before disclosure for research, the Explanatory Notes to the DEA clarify at paragraph 37:

The Act also provides for the use of de-identified (de-personalised) data to support accredited researchers to access and link data in secure facilities for the purpose of carrying out research for public benefit.

The DEA was therefore intended to provide researchers with access to linked administrative data for research of broadly defined public benefit. This could well include research investigating non-biological factors which influence or indicate public health, by linking health and non-health administrative data. However, the exclusion of ‘health’ (i.e. health service) data from that which can be shared under the research provisions of the DEA means that such research remains a challenging endeavour. This statutory exclusion means public authorities within the UK National Health Service (‘NHS’) remain reliant on the existing framework of laws already used for disclosure of information, a framework which can take years to navigate [8].

As our case studies illustrate, researchers are left with a difficult landscape when seeking access to linked ‘health’ and ‘non-health’ administrative data. Some of these challenges are specific to the particular issue of such linkage, others stem from the complexity of administrative data access in general. The ADRN was described in its Mid-Term Review Report as experiencing data access issues with ‘complex origins’ [9], and the project ended on 31 July 2018 [10]. On 27 September 2018 a new Administrative Data Research Partnership was announced between UK Research and Innovation and the Office for National Statistics, with a greater emphasis on identifying ‘shared priorities’ with government departments as a driver for research [11]. It remains to be seen what this new project will achieve, but at present it has been suggested in a report from the Office for Statistics Regulation that, despite their societal value, success stories in administrative data linkage for research remain the exception rather than the rule [12].

Added to this, the General Data Protection Regulation (‘GDPR’) has heightened awareness of the need for lawful disclosure of data, and there is some concern that its provisions relating to pseudonymisation in Article 4 [5] will make data sharing for research more difficult [13]. The obstacles to data sharing for research are not purely legal, however. It is also possible to point to cultures of risk aversion within public authorities which can compound any uncertainty in interpreting the law [12].

Even where there is a cultural will, and a legal way, linkage of health data for research requires appropriate resources, expertise and investment. Negotiation between researchers and data custodians can be dogged not only by risk aversion, but also mutual lack of understanding and replication of effort—a problem which has also been identified outside of the UK [14]. Furthermore, it must also be remembered that health data sharing is not a purely bilateral relationship, and the views of the people from whose records administrative data are derived must be taken into account. Understanding the views of an entire national population on a question in which its members will have varying levels of interest and understanding is no mean feat.

We approach this potential mass of obstacles from a multi-disciplinary perspective. Focusing on three cases studies, we delineate key issues which can prevent access to health data for public interest research and present a corresponding recommendation for each issue. We accept, as others have argued, that there is no single ‘magic bullet’ which can resolve the issues researchers face in accessing health data [15]. Instead, we explore these issues through the challenges and successes in health data sharing highlighted by our case studies, and make recommendations which reflect the multifaceted nature of the challenge.

## Approach

This paper uses national case studies to examine an issue that, despite the challenges alluded to above, the UK is well-placed to address: the potential for linked health and non-health administrative data to cast light on socio-economic, non-biological determinants of population health. The Academy of Medical Sciences has already called for more research into such factors:

*‘there remains much we do not know about the complex array of interlinking factors that influence the health of the public, and about how to prevent and solve the many health challenges we face as a population* […] *Biomedical research as currently conducted does not have the capacity to address these increasingly diverse and complex issues that transcend disciplinary, sectoral and geographical boundaries. We need to move towards a ‘health of the public’ approach… We must drive forward an ambitious research agenda to realise the aspirations of successive policymakers and leaders of health and social care — aspirations to shift our focus to prevention and early intervention at scale, and to thereby optimise the use of resources.’* [16]

The breadth of health data held by the NHS across the population should mean the UK is well-positioned to undertake this ambitious research agenda. These data are often acknowledged as a rich resource for data analytics [17], so much so it was proposed that publicly controlled data should be granted special legal protection under the Data Protection Act 2018 [18]. However, it has been recently claimed by the Office for Statistics Regulation that the value of data linkage is not currently maximised in the UK [12]. Our case studies support this claim, indicating that such research could be more widely used to determine how population health can best be served by state services.

The UK examples of health data linkage and sharing for research which we have taken as our case studies are:

1. The ADRN, the above-outlined, ESRC-funded initiative designed to provide secure linkage of, and access to, administrative data for research purposes across the UK.
2. NHS Digital, which is responsible for the national collection and dissemination of health data in England, and uses statutory powers to do so.
3. The SAIL: a safe haven in Wales which collects data on a voluntary basis from healthcare providers, and facilitates safe access to linked data for researchers.

The examples of ADRN and NHS Digital in particular illustrate the challenges of linking health and non-health administrative data, whereas SAIL demonstrates one means by which these obstacles can be successfully negotiated.

Population data science is by nature multi-disciplinary [19]; this is as true of the challenges facing access to linked data for research as it is of the ultimate analysis of such data. Two of the issues identified in this paper are legal in nature, relating to the implementation of the DEA and the GDPR. Another three, however, relate more to the infrastructure through which health data access for research is delivered across the UK, which will subsist regardless of how these pieces of legislation are construed.

To provide contextual depth to the case studies, the lead author has reviewed the literature surrounding care.data, the abandoned health data sharing programme in England which forms the background to the current data dissemination practices of NHS Digital. This is in turn contrasted with the voluntary health data collection approaches adopted in other parts of the UK [20,21], with reference to the academic literature on care.data [15,22-25], the subsequent review of health data sharing conducted by the National Data Guardian [26], and the government’s response [27]. Other reports, consultations and policy papers on data sharing have been reviewed, which point towards the future of linked health data research in the UK [8,12,16,28-30], as well as some of the issues health data sharing might face [3].

The case studies themselves have been conducted with reference to published information relating to the three organisations in question. Information relating to the ADRN, its successes [31], challenges [9], and evolution [32] are relied upon, with emphasis on information made publicly available wherever possible. The same approach has been taken with information about NHS Digital: its guidance [33], public information, and particularly its release registers [34] have been scrutinised. SAIL has, likewise, been studied from its publicly available information [35,36]. These sources of information have informed the iterative, interdisciplinary discussions between the authors, and the agreed list of issues and recommendations with which this paper concludes.

A summary of each case study is provided in the ‘Results’ section below, in which issues are identified which have wider implications for health data sharing.

## Results

### Case Study 1: The Administrative Data Research Network

While our second two case studies relate to England and Wales respectively, the ADRN was an attempt to bridge the data divisions across the UK.

The genesis of the ADRN was the report of the Administrative Data Taskforce in 2012. The Taskforce, set up by the Economic and Social Research Council, made the following recommendations:

1. An Administrative Data Research Centre should be established in each of the four countries in the UK.
2. Legislation should be enacted to facilitate research access to administrative data and to allow data linkage between departments to take place more efficiently.
3. A single UK-wide researcher accreditation process, built on best national and international practice, should be established.
4. A strategy for engaging with the public should be instituted.
5. Sufficient funds should be put in place to support improved research access to, and linkage between, administrative data [28].

While these recommendations were accepted by the government [37] and put into practice, the needs highlighted by the report six years ago remain relevant beyond the lifespan of the ADRN: particularly 2), 4) and 5).

The ADRN was duly set up with four Administrative Data Research Centres (England, Wales, Scotland and Northern Ireland) to facilitate access to administrative data for approved researchers. Where researchers required linked administrative data a ‘Trusted Third Party’ model was used, in which the identifiable data required for linkage are separated from the sensitive health or service data required for research. Under a Trusted Third Party model, no one individual is privy to identifiable sensitive data that they do not already hold. The confidentiality of the information is protected by separating the identifying information (e.g. names) from the substantive information about the individuals in question, i.e. the content of their records.

Despite the statutory uncertainty surrounding health data linkage for research, it is evidently not impossible. The 106 projects listed on ADRN’s featured research page [31] include sixteen studies using linked data from NHS Digital, and nine which use such data for research which does not focus exclusively on the health service, for example:

- An evaluation of how special education needs provision for children with Down syndrome impacts upon emergency hospitalisations [38];
- An exploration of the links between job characteristics and health [39];
- Development of an Index of Multiple Deprivation; identifying the area most in need of public investment [40];
- Exploring the relationship of education and health outcomes for children and young people in England [41].

That said, many of these projects required protracted negotiations for data access which would not have been possible without a significant commitment of researcher time. The last project listed, for example, required 3 years, 11 months, 6 meetings and at least 108 email and telephone correspondences to gain approval for linkage of the required data, against a backdrop of a shifting legal landscape [42]. Access proved an issue across the ADRN as a whole, particularly where the data in question were requested from a central government department. The ADRN Mid-Term Review Report found that there was *‘near-unanimous agreement that lack of administrative data from government departments is the single biggest challenge that the Network faces’* [9]. While uncertainty surrounding interpretations of powers to share data was not the sole cause of this difficulty, it was experienced as a significant factor by those involved.

**Issue 1: The lack of a clear legal route for linking health and non-health administrative data, even when research using such linked data might benefit public health.**

In response to the difficulty of accessing administrative data, Thematic Partnerships were established to focus research efforts on a few, large datasets to which the Network could negotiate access [32]. The ADRN acknowledged that its ad hoc pursuit of linkage projects, as requested by individual researchers, was not an efficient way of effecting impactful research. Long delays in negotiating access to data had to be justified by a ‘high-yield’ result, ideally a result which could be used by multiple projects to maximise the potential public benefit from the time and resources spent on achieving the linkage. The original model of the project was therefore abandoned in its latter stages. The ADRN project came to an end on 31 July 2018 [10], with the scale of its original ambitions recognised as not realised in full owing to difficulties in accessing data [12]. It has since been succeeded by the Administrative Data Research Partnership, which furthers the model of thematic partnerships and planned programmes of linkage.

A review of the featured research suggests that the majority of ADRN-supported research did not cross national boundaries. The Me-D-Links study, approved in July 2016, is described as *‘one of the first, if not the first, projects conducted within the ADRN using data from all 4 regions of the UK’* [43]. The issue of linking health and administrative data across national boundaries within the UK may be a point on which further work is needed. If the UK cannot harmonise its governance for health data sharing, more ambitious projects using information from beyond the UK are a far-off prospect.

**Issue 2: Ad hoc linkage is less efficient than planned linkage projects, and data governance could be better harmonised to support cross-centre sharing, particularly across national boundaries.**

### Case Study 2: NHS Digital

‘NHS Digital’ is the working name of the statutory Health and Social Care Information Centre (‘HSCIC’), a name adopted following the controversy surrounding the care.data programme. The original stated aims of the care.data programme were entirely laudable: supporting patient choice, advancing customer services, promoting greater transparency, improving outcomes, increasing accountability and driving economic growth by making England a centre for world-class health services research [44]. However, the programme encountered significant issues in its implementation, issues which led to its ultimate abandonment. It was intended to achieve its aims by bringing health and social care data in England together under the governance of the newly established HSCIC, for dissemination to researchers in pseudonymised form.

The time frame in which care.data was originally intended to be implemented proved problematic. Academic commentators emphasised the need for adequate consultation to satisfy the law of confidentiality [45]. General Practitioners were given eight weeks to inform patients that data would be provided to the new HSCIC. This prompted an outcry from GPs [25].

A subsequent leaflet campaign intended to make good the informational deficit was threatened with a letter before action [46]. The programme was paused, reviewed by the National Data Guardian (who supported re-naming the HSCIC to emphasise its status as part of the NHS ‘family’ [26]) and finally abandoned in July 2016.

The HSCIC was renamed NHS Digital from July 2016 in order to build public confidence and trust [47]. NHS Digital continues to collect and disseminate health data in England. The overwhelming majority of the releases have been made to other NHS organisations or local authorities, alongside a smaller number of releases to ‘for-profit’ entities and university researchers.

Anonymisation plays a key role in NHS Digital’s dissemination of health data for research. A review of its release registers [48] for December 2016 to May 2018 suggests that between 76% (3352/4410 in December 2016—February 2017) and 86% (5238/6107 September-November 2017) of releases were justified on the basis of anonymisation to the standard set by the Information Commissioner’s Office (‘ICO’) [49]. There is also evidence that the public prefers health data shared for research to be ‘anonymous’ [50], meaning that such processing might be helpful for engaging public trust or confidence in data sharing. However, it is not clear what members of the public understand by the term ‘anonymous.’ The word could be interpreted, for example, as meaning a type of data in which identification of individuals is impossible in any context; this is, according to the UK Anonymisation Network (‘UKAN’), not possible as anonymity cannot be assessed independently of environment [51]. The reality of what is required by the ICO is data which do not give rise to a reasonable likelihood of identification in the context of a particular environment; an environment which could change and must be kept under review [49]. Anonymisation is therefore not a complete solution to the problem of securing trust and confidence in dissemination of health data: the public does not necessarily have a clear picture of the persisting risks of identification, and management of such risks can be complex—hence why the UKAN Anonymisation Decision-Making Framework has 10 components [51].

The accompanying statutory power for these ‘anonymised’ disclosures is listed in the release registers as the Health and Social Care Act 2012 (‘HSCA’). As the most recent register (May-September 2018) has clarified, this means s.261 HSCA [48], which contains a provision which limits the ‘why’ of disclosure (i.e. the purpose for which data can be shared), but not the ‘who’, i.e. the types of people or organisations with whom they can be shared. Consequently, a number of different types of public and private organisations have received ‘anonymised’ data under this provision, on the basis that their requests were deemed to be for the purpose of health or social care, or for the promotion of health.

Where data are not anonymised, authorisation of unconsented use of identifiable health data is usually processed via recommendations from the Health Research Authority’s Confidentiality Advisory Group ‘CAG’. This group typically meets once or twice a month [52] to assess applications, and published case studies suggest that applying for CAG approval can be a lengthy process [11]. If obtaining data through this route is already challenging for some researchers, it is difficult to envisage how this system could deal with the number of access requests currently processed via ‘anonymisation’ of the health data. As such, it is troubling that there are suggestions that the administrative data in general [13], and health data in particular [53], will become more difficult to anonymise and share under the GDPR.

**Issue 3: Existing challenges in processing health data for research could be compounded by the uncertainty surrounding the GDPR.**

Under its Service Level Agreement, NHS Digital commits to response times of 14 working days for standard requests; 30 for ‘medium’ requests and 60 working days for more complex requests. However, the experience of many researchers is that these timescales can be significantly exceeded. This can be particularly challenging for publicly funded academic researchers, who often work to tight funding deadlines. As the 2017 Life Sciences Industrial Review highlighted, where linked data are requested, multiple (independently considered) applications may be required, and the involvement of the CAG can lead to significant delays [8].

**Issue 4: The delay and uncertainty of timescales of health data access for research is a problem, especially for publicly funded academic researchers.**

### Case Study 3: SAIL

In Wales, access to a wide range of health datasets has been greatly improved through the establishment of SAIL. SAIL operates as a secure safe haven for anonymised health data in Wales. As within the ADRN, a Trusted Third Party model is used for linkage, although in the case of SAIL the Trusted Third Party is the NHS (Wales) Informatics Service, so confidential patient information remains within the health service. Linked data are then accessed by researchers via the SAIL Gateway, a remote access technology and analysis platform enabling approved researchers to access data within the SAIL virtual environment from their own desktop anywhere in the world. The protocols in place allow user authentication and monitoring, and prevent the alteration or removal of SAIL data by users.

This model has proved to be a highly successful development both in terms of data security and data access, such that this infrastructure improves health data accessibility for research whilst maintaining confidentiality. SAIL can reduce the time traditionally taken to provide access to data to prevent health research being delayed or abandoned. The approvals process is designed with researchers in mind, so that scoping may be done before funding is requested, and the average time for Information Governance Review Panel approval to access data is 12 weeks, including time taken to undertake any necessary Safe Researcher Training.

SAIL also offers an example of successful public engagement to support health data sharing. A 2013 report by the Organisation for Economic Co-operation and Development commended its multifaceted efforts to engage with stakeholders, including public representation via a consumer panel and steering groups [54]. The public engagement work undertaken by SAIL is clearly instructive, and worth taking into account as a precedent. Since 2011, SAIL has had a thriving and active Consumer Panel comprising members of the public with a variety of interest areas. The Panel advises SAIL on data protection issues in data-intensive research from the perspective of service users and carers. Researchers are encouraged to meet with the Panel to discuss their proposals and receive a public viewpoint and advice; as such, SAIL can support individual projects to conduct appropriate engagement on their proposed use of administrative data.

The Office for Statistics Regulation contrasts SAIL’s ‘tandem’ approach with the top-down evolution of NHS Digital, who (they suggest) are not entirely confident of their social licence to share data [12]. Another difference is that SAIL involves researchers and methodologists who understand how data are used for research, whereas NHS Digital is separate from academia. The gradual development of SAIL in collaboration with various stakeholders compares positively with the history of care.data from the previous case study. It cannot be assumed that the same engagement model can be adopted for every project, or even for data-sharing infrastructure. There is no ‘cut-and-paste’ model of engagement that we would endorse in all situations, regardless of the source of the data, the operational model of the research or the particular sensitivities of the affected groups. However, the process by which SAIL’s engagement model was developed—gradually, and with time for feedback from data providers, the public and the research community to be taken into account—is a helpful precedent for future data-sharing infrastructure, and in particular structures set up to share administrative data (health or non-health) for research. This further underscores the importance of planned programmes of data linkage, which allow time and resources for the development of appropriate engagement mechanisms.

**Issue 5: Securing and maintaining the social licence for administrative data sharing remains a challenge.**

### Recommendations & Discussion

In light of the above case studies, and the issues they highlight, we propose the following recommendations:

#### 1) A clear legal route should be identified to enable linkage of health and non-health administrative data for public interest research.

This recommendation relates to the first issue identified within the ADRN case study: the lengthy negotiations which can result from uncertainty around legal powers to link and share administrative data for research.

In England, the NHS Act 2006 governs the use of identifiable patient information for ‘medical purposes’, which is defined as encompassing ‘medical’ research [55]. It is this provision, with its requirement for research to be ‘medical’, which is the most commonly used ground for processing identifiable data for research linkage where data subject consent is not practicable [56] (as is usually the case where administrative data are used for research, as evidenced by the NHS Digital release registers). In its current published standards, NHS Digital indicates that de-identified data should be used for purposes other than direct care, such as epidemiology [33]. In such cases access requests must usually satisfy the broader (if not entirely uncontentious) requirement of being for the provision of health care or adult social care, or for the promotion of health, under s.261 HSCA.

Researchers seeking to study (for example) the optimisation of non-health services for health benefits run the risk that their research will be deemed non-medical, and will thus find it difficult to demonstrate how the identifiable data can be lawfully processed for linkage. Detailed guidance as to the exact scope of ‘medical purposes’, and indeed medical research, is limited, but the Health Research Authority gives the example:

*‘requests to access patient information to inform road traffic management planning could not be approved as the primary purpose would not support health service improvements.’* [57]

This is interesting, as it suggests that planning services other than the health service goes beyond the ‘medical’, even where such services impact upon health. For example, a study of the health impacts of building busy roads near primary schools could be deemed to be out of scope, however pertinent the public health concerns it would address.

The DEA could have been the answer to this ‘medical purpose’ quandary, but health data were excluded after representations were made about the need to protect patient confidentiality [58], even though the Act does not allow identifiable data to be disclosed to researchers (only for it to be processed securely, de-identified and then disclosed). As the DEA has now been debated and passed with the exclusion, however, it remains to be determined how health and non-health administrative data can be lawfully linked for research. The well-documented pressures on the NHS demand that research enabling optimisation of other services for better public health be properly supported.

A review of the legal options suggests a number of possible routes:

1. NHS Digital could disclose information to the Office for National Statistics using existing legal powers (e.g. s.45A Statistics and Registration Service Act 2007); the Office for National Statistics could then link these data and share them in a de-identified state under the DEA, or under s.39 [4] of the Statistics and Registration Service Act.
2. It could be clarified whether the scope of ‘medical research’ can include research using non-health data. This could be research which uses non-health data to evaluate healthcare (e.g. using disability benefit data to assess clinical outcomes), or which links such data with medical records to measure the health impacts of public policy, or to study important socio-economic determinants of health.
3. NHS Digital could act as a Trusted Third Party for other public authorities, receive data, link them with health data and disclose them to researchers in de-identified form for the promotion of health under s.261 HSCA.

It must be stressed that we are not recommending new grounds on which identifiable data can be provided to researchers, but simply to enable the processing of identifiable data for linkage in a secure environment for analyses of de-identified data. Whatever the route, updated guidance from (for example) the Health Research Authority would help to clarify the circumstances in which health data can be linked with other types of data for research, and the appropriate scope of ‘medical research.’

It is hoped that that NHS Digital, and those working to implement the research provisions of the DEA, will find an appropriate route to link health and non-health administrative data. If no point of intersection between the statutory regimes can be found, legislative amendment may be necessary for ‘joined-up’ data to be routinely available.

#### 2) Research data providers should adopt planned approaches to linkage, based on privacy by design principles.

The decision within ADRN to focus on a few high-value datasets, for which linkage is feasible, is comparable to international attempts to construct routine ongoing linkage systems [59], as well as to SAIL’s approach to linkage. In each case, resources are allocated to identifying datasets that have a high research value that would be enhanced through linkage to other datasets, and linkage is then performed to support a range of unspecified future research projects.

A prospective, proactive, or ‘planned’ approach to linkage means governance issues surrounding identifiable data linkage can be addressed separately from the question of anonymous or pseudonymised data sharing for research. The legal, political and technical aspects of data linkage can take many years to navigate, while the task of processing requests for de-identified data that have already been linked is comparatively easy. In carrying out planned linkage, high-value linkages (e.g. those likely to support multiple research projects) are identified and data providers can focus on the question of whether they have the legal power to carry out the linkage, identify a lawful pathway (should there be one), develop technical solutions for implementation (involving a third party if necessary), and then begin the process of linkage. The question of whether anonymised extracts can then be shared with a specific applicant for a specific application can then be addressed as a subsequent process, with much less delay to researchers.

This approach is resource-efficient and provides opportunity to reduce and isolate one of the biggest components in the cost barrier to research using linked data. It lends itself readily to incorporation and monitoring of systems for safe data storage and access (e.g. safe havens). It allows for expertise in linkage to accrue and the quality of linkages to be refined over time. It also means that data custodians have the opportunity to provide well-signposted routes to data access and dataset listings so researchers can see what is available and how to get there.

For planned linkage to be truly far-sighted, it would be prudent for data controllers to operate along consistent principles of data governance. The GDPR contains a broad stipulation that data protection principles should be built in ‘by design and by default’ when processing personal data, but does not provide specific guidance as to how to operationalise this requirement. While the GDPR’s ‘privacy by design’ requirement provides a useful starting point for research data providers, further work could be done to harmonise implementation of key features of this central requirement, such as how ‘data minimisation’ is interpreted when data are linked with a view to retention and multiple research use.

For example, the GDPR has broad requirements of appropriate data security and record keeping, but exactly how to operationalise these obligations is left to individual organisations. If similar technical and organisational measures are adopted across data-holding bodies, these entities are better placed to share data with each other, as each can offer broadly equivalent levels of protection of the data. Consistent, privacy-preserving principles could thus help facilitate data sharing and cross-centre working.

A greater level of data sharing within the different nations of the UK would build upon what the ADRN began to achieve within its five-year tenure.

#### 3) Guidance on health data sharing under the GDPR should be proportionate to identification risk.

Our third recommendation is drawn from the NHS Digital case study, which highlights both the importance of anonymisation for the dissemination of linked health data in the UK, and the potential for the GDPR to disrupt current systems for data sharing.

NHS Digital is heavily reliant on the ICO’s standards for anonymisation. Under these standards, the majority of the releases to researchers in December 2016-September 2018 have been deemed to be anonymous. If this significant majority of releases were re-interpreted as disclosures of personal data, this could require large-scale authorisation by CAG (or some alternative body nominated by the Secretary of State for Health). Aside from the considerable resource and infrastructural challenge this would pose, there is also a risk that research of real public interest would be excluded on the grounds that it does not meet a narrow definition of ‘medical’ research (see recommendation 1).

At the time of writing, the UK ICO has yet to release their updated guidance on anonymisation under the GDPR. They have indicated that their existing, pre-GDPR guidance is ‘a good starting point’, suggesting that this guidance (used by NHS Digital as a benchmark for disclosure) can still validly be followed under the GDPR [60]. However, until this updated guidance is released, a degree of caution is justified.

The crux of the matter is the relationship between anonymisation and pseudonymisation under the GDPR. The GDPR clearly defines pseudonymisation in Article 4 [5] as a process by which personal data are ‘minimised’ (i.e. rendered less identifiable) but nonetheless remain personal. For an organisation such as NHS Digital which pseudonymises data but holds the original identifiers, these identifiers are a ‘means reasonably likely to be used’ to identify individuals. Therefore, they would be deemed to be processing personal data when using pseudonymised data.

What are less clear, however, are the conditions under which data that have been pseudonymised can become anonymous when shared with a third party who has no means of identification (such as a researcher). A reasonable interpretation would be that if the researcher, as a member of another organisation, does not have access to the identifiers, or any other means reasonably likely to be used to identify individuals, then by definition individuals are not ‘identifiable’ and the data should not be considered personal for the researcher’s organisation. The ICO’s updated guidance is potentially supportive of this interpretation:

*‘In order to be truly anonymised under the GDPR, you must strip personal data of sufficient elements that mean the individual can no longer be identified. However, if you could at any point use any reasonably available means to re-identify the individuals to which the data refers, that data will not have been effectively anonymised but will have merely been pseudonymised. This means that despite your attempt at anonymisation you will continue to be processing personal data.’* [60]

The emphasis on ‘you’ appears still to be directed at an individual organisation. If ‘you’ as a data user do not at any point have any means reasonably likely to be used to identify the data, the data could be deemed non-identifiable.

The only caveat to this interpretation is the question of how NHS Digital, or any other data provider, gains sufficient assurances that a researcher would not have a means reasonably likely to be used to identify individuals. This was the case before the GDPR, as the distinction for personal or anonymous data was essentially the same: can the controller in question (or anyone else who has access to their data) identify individuals by any means reasonably likely to be used? The challenge post-GDPR is still to determine whether reasonably likely means of identification have been excluded, and therefore whether data can be called anonymised or merely pseudonymised. What is uncertain, however, is whether the ICO uses its updated guidance to introduce new and more stringent standards as to when shared data can be called ‘anonymised.’ We cannot discount this possibility, given the time the ICO has taken in the revision and the concerns cited above.

Where data have been linked, and then shared for research, the pseudo-identifiers themselves are not necessarily the issue, as these can easily be generated randomly and separately for each project. The question relates more to what other data sources may be combined with the payload research data. For example, access to identifiable information about some people’s health histories (even the researcher’s own history) would potentially allow their re-identification in hospital data using information about dates and types of treatment.

In the case of ADRN, a careful data situation audit was conducted of the controlled environment in which researchers accessed data [51]. SAIL relies upon a suite of controls including Safe Researcher Training, data access agreements, and a secure Virtual Private Network for researchers to access prepared datasets, to control how data are used and minimise identification risk [61]. Thus far, NHS Digital has not altered in its reliance on ‘anonymised’ releases to researchers post GDPR, as the majority of its May-September 2018 releases are still defined as ‘Anonymised–ICO code compliant’[48]. What remains to be seen is what impact, if any, the new UK Anonymisation Code has on these significant numbers of releases currently disseminated on the basis they are ‘anonymised.’ The implementation of the GDPR may mean that safe havens such as SAIL or ADRN, which have the resources to exercise scrutiny over (re)identification risk, are all the more important to the lawful sharing of health data for research.

Clearly, work is needed between health data providers and regulators. The ICO’s pre-GDPR guidance on anonymisation states that determination of whether data are personal calls for ‘sensible judgement based on the circumstances of the case in hand’ [49]. It is hoped that this sense of proportionality is maintained in the dissemination of health data for research, even under the GDPR, so that public interest research is not impeded.

#### 4) Organisations providing health data for research should have in place clear, transparent and efficient response times.

Building on one of the recommendations of the 2017 Life Sciences Industrial Review [8], organisations linking and disseminating health data for research—be they public authorities or independent safe havens—should provide clear, transparent and reasonable timescales for researchers wishing to access linked data. Additionally, we suggest that the management of data access requests for academic research should be delineated from the management of other types of request, with timescales for academic applications reported separately, and guidance made available to academic researchers on how to demonstrate the necessary benefits in their application.

The example of NHS Digital illustrates how academic requests for data access can suffer when processed alongside operational applications. Applications from other public authorities have been streamlined [62,63], and private companies may have sufficient resources to play the long game [64]. For academic researchers working to tight funding deadlines, however, delay can be fatal to a project. Research grants typically provide between one and five years’ funding, and require research activities to be costed in detail at the outset. Nevertheless, costs for data provision and linkage are often not quoted until after many months, sometimes years, of work has been poured into applications.

Publicly funded researchers need reasonably foreseeable timescales when they apply to access health data: we suggest that transparency, as well as efficiency, is key to achieving this aim. Data applications for academic research form a small minority of applications: a review of NHS Digital’s Data Releases from July 2015 to February 2017 conducted by one of the authors suggests about 4% of these releases were made to universities. It should be possible for data providers to report timings separately from application to approval for this type of applications, and thus provide the foreseeability academic researchers require.

Academic requests may also suffer when considered alongside those with a more operational focus because their benefits can be more difficult to define in the short term. The benefits of research for the health service are inevitably uncertain; they depend on the nature of the research findings, and how other actors (e.g. policy makers) respond to them. In contrast, service commissioners, local authorities or commercial companies often use data for pre-specified monitoring, accounting, or for creating dashboards to show how a service is performing. Thus a one-size-fits-all application system fails to address these very different contexts, and researchers can be required to be unrealistically definitive about benefits. Delineation of academic applications from other types of data access request could help to focus on the particular requirements and benefits of academic uses of health data.

As a minimum, clarity and consistency of timescales for data access is a must for publicly funded research, which is never free of its own mandatory deadlines. This may be easier to achieve where linkage is carried out in a planned way (see recommendation 2).

#### 5) Public engagement should be ‘by design and default’ in systems for health data sharing, including accessible information on data sharing, and promotion of the value of health data research to the NHS and to society.

The contrast between the evolution of SAIL and of NHS Digital highlights not only the importance of engagement with key publics, but also the need to develop an engagement strategy suitable to the project or infrastructure in question.

Public engagement and involvement must be not only recognised as important, but also as requiring tailoring to the project in question—for example, depending on the data which are to be used and the proposed operational model of the research. There is developing nuance within the literature surrounding public attitudes towards health data linkage for research. A 2016 systematic review has revealed widespread, but conditional, support for data linkage and sharing in health research [50]. These conditions have been further explored in a discrete choice experiment, which stressed that (for example) the type of data in question, i.e. what kind of information it contained and where it originated from, was a far more important factor to respondents than the type of researcher (academic or commercial) accessing the data [65]. It cannot be assumed, therefore, that patients will feel equally supportive of all academic research, and projects using cross-sectoral linked data may require a more rigorous consideration of public acceptability than those exclusively using linked health data.

We accept, however, that there may be common principles which underpin the development of a public engagement and involvement strategy. An international consensus statement has emphasised transparency, inclusivity, clarity of purpose, designed to produce impact and ability to be evaluated as core principles within public involvement and engagement in research [66]. These principles echo the themes which arise from our own recommendations (see below). We therefore suggest that these principles be taken into account when developing engagement strategies, and refer readers to this consensus statement for further detail. The development of a strategy will involve a selection of the appropriate mechanism, or combination of mechanisms, to engage with stakeholders and the wider public; for example a public panel (such as that used by SAIL [67]), exploratory workshops, a more intensive ‘co-design’ model, or a more information-based campaign which provides transparency without necessarily seeking feedback.

While individual projects may be able to engage successfully with small numbers of people (e.g. a patient panel), a high-impact dissemination of information is more feasible on a national scale. The fact that ADRN particularly struggled to access UK government data suggests that NHS Digital may not be unique in its concern about social acceptance of data sharing for research [12]. Ultimately, a high-profile engagement campaign from institutions such as the Department of Health (as promised, at least to an extent, in the response to the National Data Guardian’s review of opt outs [27]) is needed to remedy any informational deficit. The patient population must be made aware of the risks and benefits of the use of linked health data for research, in order to secure the ongoing public acceptance of this important area of work.

## Conclusion

The themes which emerge from our recommendations are clarity, transparency and efficiency. Clarity as to legal powers to link public sector data—and the limitations created by data protection law—could be achieved through updated guidance. Clarity from data providers as to timescales for data access, and how to demonstrate appropriate public benefit in research proposals, would assist researchers, as well as efficiency in data request management. Meaningful transparency with the patient population is also essential, and should be informed by careful, evidence-based reflection.

In light of the myriad legal, political and social issues which must be considered prior to linkage of public sector data, we advocate a planned approach. If data of high value for research is identified for linkage, with a view to making it available to appropriate projects at a later date, there is time to clarify legal issues, provide transparent frameworks for researchers, and inform or involve patient groups as appropriate. Despite the challenges faced by ADRN, investment in planned linkage holds much promise as a means to overcome the key obstacles to health data linkage. It is hoped that such approaches will be adopted more widely, so that the public interest potential of linked health data in the UK does not remain an under-utilised resource.

## Abbreviations

ADRN	Administrative Data Research Network
CAG	Confidentiality Advisory Group
DEA	Digital Economy Act
GDPR	General Data Protection Regulation
HSCA	Health and Social Care Act 2012
HSCIC	Health and Social Care Information Centre
ICO	Information Commissioner’s Office
NHS	National Health Service
SAIL	Secure Anonymised Information Linkage Databank
UK	United Kingdom
